# Continuous table tennis is associated with processing in frontal brain areas: an EEG approach

**DOI:** 10.1007/s00221-022-06366-y

**Published:** 2022-04-25

**Authors:** Anton Visser, D. Büchel, T. Lehmann, J. Baumeister

**Affiliations:** Exercise Science and Neuroscience Unit, Department Exercise and Health, Paderborn University, Warburger Str. 100, 33100 Paderborn, Germany

**Keywords:** Exercise neuroscience, Sports, Physical activity, Rehabilitation

## Abstract

Coordinative challenging exercises in changing environments referred to as open-skill exercises seem to be beneficial on cognitive function. Although electroencephalographic research allows to investigate changes in cortical processing during movement, information about cortical dynamics during open-skill exercise is lacking. Therefore, the present study examines frontal brain activation during table tennis as an open-skill exercise compared to cycling exercise and a cognitive task. 21 healthy young adults conducted three blocks of table tennis, cycling and n-back task. Throughout the experiment, cortical activity was measured using 64-channel EEG system connected to a wireless amplifier. Cortical activity was analyzed calculating theta power (4–7.5 Hz) in frontocentral clusters revealed from independent component analysis. Repeated measures ANOVA was used to identify within subject differences between conditions (table tennis, cycling, n-back; *p* < .05). ANOVA revealed main-effects of condition on theta power in frontal (*p* < .01, *η*_p_^2^ = 0.35) and frontocentral (*p* < .01, *η*_p_^2^ = 0.39) brain areas. Post-hoc tests revealed increased theta power in table tennis compared to cycling in frontal brain areas (*p* < .05, *d* = 1.42). In frontocentral brain areas, theta power was significant higher in table tennis compared to cycling (*p* < .01, *d* = 1.03) and table tennis compared to the cognitive task (*p* < .01, *d* = 1.06). Increases in theta power during continuous table tennis may reflect the increased demands in perception and processing of environmental stimuli during open-skill exercise. This study provides important insights that support the beneficial effect of open-skill exercise on brain function and suggest that using open-skill exercise may serve as an intervention to induce activation of the frontal cortex.

## Introduction

Physical exercise does not only improve fitness but is further able to enhance cognitive functions (Chang et al. 2012). Several findings assume that—apart from exercise intensity—the modality of the exercise performed may modulate the beneficial response of the brain to exercise (Voelcker-Rehage et al. 2010; Tsai et al. 2017). Cross sectional studies suggest that challenging coordinative exercise, where the individuals are asked to actively engage within the environment, seems to induce a more beneficial effect on cognitive functions in terms of inhibitory control (Wang et al. 2013a), temporal processing (Wang et al. 2013b), task switching (Yu et al. 2017) or perceptual processing (Zhou et al. 2020) compared to exercise with restricted coordinative demands or cardiovascular exercise only. A potential stimulus for these superior adaptations are the highly cognitive and multisensory demands of coordinative exercise (Wang et al. 2013a).

The requirements of coordination exercise may change when performed in an unpredictable environment referred to as open-skill exercise (Schmidt and Wrisberg 2004). Sports like, badminton, table tennis (TT), tennis and team sports are characterized by everchanging stimuli requiring subsequent adaptation of movement to the dynamic environment (Gu et al. 2019; Ingold et al. 2020; Zhou et al. 2020). For instance, during TT it is essential to track the movement of the ball, anticipate the trajectory, react correspondingly and finally return towards the opponent’s court (Akpinar et al. 2012). Accordingly, this complex game interaction requires visual attention, decision making and action execution (Taddei et al. 2012). Thus, the continuous demands in perception and processing of environmental stimuli for subsequent movement adaptation as a key element of open-skill exercise are suggested to particularly elicit the beneficial effects of open-skill sports on brain function (Gu et al. 2019).

Recent technical developments in mobile brain imaging open new opportunities to monitor the contributions of the brain during the actual performance of a given full-body motor task in rifle shooting (Luchsinger et al. 2016), golfing (Baumeister et al. 2008) or running on a treadmill (Gwin et al. 2011). To investigate brain contribution to sports performance, electroencephalography (EEG) applications outperform near-infrared spectroscopy regarding temporal resolution and functional magnetic resonance imaging regarding mobility and temporal resolution despite of limitations in spatial resolution (Mehta and Parasuraman 2013). Therefore, EEG allows to investigate human brain dynamics in real-world interactive environments (Baumeister et al. 2010; Reinecke et al. 2011).

Using EEG, an applicable approach to quantify continuous attentional processing during motor tasks is the analysis of power spectral density (Büchel et al. 2021; Gebel et al. 2020; Anders et al. 2018; Cheron et al. 2016). An overall increase of activation during TT in motor-related areas of the brain has been shown for both novices and experts utilizing functional near-infrared spectroscopy (Carius et al. 2021; Balardin et al. 2017). The power spectral density analysis of EEG data can expand these findings by revealing the activity within specific frequency bands. In stationary experiments, theta oscillations (4–7 Hz) in frontal brain areas seem to play a crucial role in continuous cognitive functions such as attentional processing, decision making, and executive function (Klimesch 1999; Sauseng et al. 2010). Based on power spectral analysis, increased activity in theta frequency is associated with higher levels of attentional focus (Baumeister et al. 2008; Luchsinger et al. 2016) and continuous error monitoring (Hülsdünker et al. 2015; Gebel et al. 2020; Anders et al. 2018) during challenging coordination exercise. Therefore, frontal theta power might be a valuable biomarker of attentional demands induced by the perception and processing during coordination exercise.

While few studies investigated cortical activation during stationary cardiovascular exercise and majorly reported broadband changes in brain activity independent from frequency band and brain region (Ghorbani and Clark 2021; Robertson and Marino 2015), there is a lack of studies investigating the activation of the human brain during continuous open-skill exercise. Nevertheless, the variable environment in open-skill exercise may elicit continuous behavioral adaptations which require increased attentional demands (Wang et al. 2013a). Especially processing of complex sensory information and sensorimotor integration, which supports voluntary behavioral responses, are associated with frontal brain lobe activity (Amaral and Strick 2013).

Due to methodological challenges, recent studies investigated subcomponents of open-skill exercise with simplified subject-environment interactions. Instead of requiring the participant to adapt movement from trial to trial, the investigations analyzed cortical dynamics of small fractions of the actual open-skill exercise. For instance, tasks like badminton backhand serves (Skrzeba and Vogt 2018), basketball free throws (Chuang et al. 2013), stationary ice hockey shots (Christie et al. 2019) were performed in a stable environment without unpredictable task elements. Beyond that, first studies use variable stimuli requiring participants to respond to three different, unpredictable ball trajectories in a TT serve-response paradigm (Hülsdünker et al. 2020), which approximates the demands on perception and processing of environmental stimuli towards the real world demands in TT (Akpinar et al. 2012). While these investigations majorly focused on movement and associated motor-evoked potentials, the element of continuous perception and attentional processing of unpredictable environmental stimuli as a core element of open-skill exercise seems to be underrepresented in sports neuroscience research.

Therefore, the present study aims to explore the underlying activation of the frontal cortex during continuous open-skill exercise. To depict cortical dynamics induced by unpredictable environments, table tennis was chosen since it demands visual attention, decision making, and action execution induced by a fast-changing environment (Akpinar et al. 2012).

From a neurophysiological perspective, table tennis is associated changes in brain activity in sensory (Hülsdünker et al. 2017; Wolf et al. 2014) and frontal brain areas (Guo et al. 2017). Similar to the increased neural activation in motor and premotor areas of experts compared to novices in table tennis (Carius et al. 2021), experts revealed higher frontal theta power compared to novice (Baumeister et al. 2008) in golfing. Consequently, repeated acute increase in frontal brain activation induced by table tennis may serve as a stimuli for long-term adaptations. According to the neural efficiency hypothesis, athletes brains seem to demonstrate task-related reorganisation of neural networks leading to increased behavioral performances (Guo et al. 2017; Del Percio et al. 2009).However, there is a lack of insights into cortical processing from frontal brain areas in an open skill task using table tennis as a model.

It is assumed, that (1) open skill exercise spawns increased patterns of cortical activation compared to (2) cardiovascular exercise in a predictable environment. For purposes of internal validation of increased attentional processing, frontal theta power during open-skill exercise will also be compared to (3) a solely cognitive task requiring working memory (Scharinger et al. 2017). Thus, TT specific frontal cortex responses will be compared to both ergometer cycling (EC) as well as the n-back task (NB). Evidence for enhanced activity of the frontal cortex during TT play as a model of open-skill exercise may contribute to a better understanding of long-term adaptations due to coordination exercise (Voelcker-Rehage et al. 2010).

For instance, TT like exercise may allow to improve cognitive function in children (Becker et al. 2018) and healthy adults (Chueh et al. 2017) and also to develop tailored interventions to prevent cognitive decline in the elderly (Huang et al. 2014).

## Methods

### Ethics

The study was approved by the Ethics Committee of the Paderborn University and was designed in accordance with the Declaration of Helsinki. Written informed consent was obtained from all participants of the present study.

### Participants

In total, 21 healthy novices in TT volunteered in this study. All participants met the eligibility criteria of age (18–30 years), handedness (right-handed), and expertise (novices by means of no active membership and less than 3 year experience in racket sports). One participant was excluded from the study due to technical problems with EEG acquisition. Two participants were excluded, since they were not able to meet the demands of the NB represented by extraordinary high non-responding rates (> 30%). Finally, 18 participants remained for data analysis (8 ♀, 10 ♂, 24.72 (± 3.32) years).

### Procedures

**Fig. 1:**
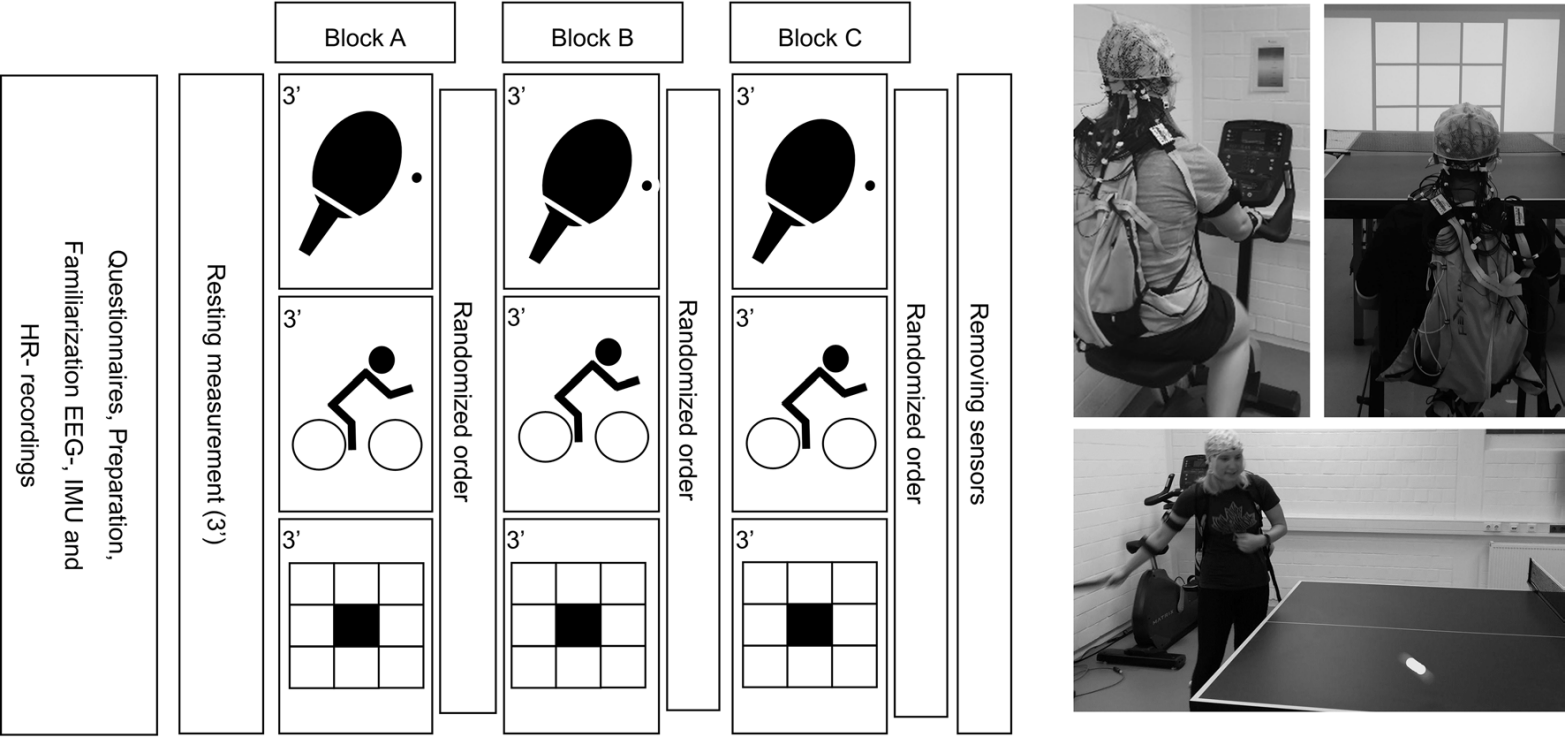
Overview of the experimental procedure. After questionnaire assessment and a 3-min resting state electroencephalography (EEG) measurement, three blocks of table tennis, cycling exercise and n-back task were performed in a randomized order

After arrival at the laboratory facilities, the EEG cap was applied to the participants head and inertial measurement unit sensors (IMU, Myo Motion, Noraxon, Scottsdale, United States) were attached to the right lower and upper arm, and the upper thoracic spine to track the motion induced by TT play. Additionally, a heart rate sensor (Polar H10, Kempele, Finland) was applied to continuously monitor the heart rate (HR). Throughout the experimental procedure, participants performed three blocks of TT play, EC and a working memory NB in sets of three minutes each in randomized order. An overview of the experimental procedures including pictures from the experimental setting is provided in Fig. 1.

Participants performed continuing TT rallies with one investigator using a standard-sized table (size: 274 by 152.5 cm height: 76 cm) and standard TT rackets and balls. Participants and investigator were asked to play cooperatively and instructed to continue a rally after the investigator’s serve for as long as possible. The investigator adjusted ball speed, slope and direction according to the participants playing skill. In case of a miss or mistake, a new ball was immediately served by the investigator. Participants were allowed to move freely but asked to avoid intense body motion, jumps and intense hits, since these may contaminate the EEG signal with non-stereotype artefacts. In case of extended artefact contamination during a rally, the participants were informed about this after a block and asked to move less intense in the next block. The number of hits per rally was approximated by analyzing the kinematic information based on the IMU sensors. The used IMU system was previously validated in the context of movement analysis (Berner et al. 2020). Thereby, the positive peak deflections in the degree of right shoulder flexion were counted to approximate the number of hits per block. The number of interruptions per block was not counted.

For EC, participants were asked to exercise at a stationary cycling ergometer (Matrix Fitness, Cottage Grove, USA) for three minutes at an individual pace. Applying a visual analogue scale according to Borg ranging from 6 (“no exertion at all”) to 20 (“maximally hard”), subjects were asked to cycle at a low to moderate cardiovascular intensity corresponding to a subjective rating of perceived exertion (RPE) around 11 (“easy”).

For NB, participants were asked to perform a visuospatial three-back task sitting in front of a wall displaying continuously changing stimuli within a 3-by-3-grid (Jaeggi et al. 2010). In total, 90 stimuli were presented, affording 87 responses per NB set. Due to inter-individual differences in processing capacity (Jaeggi et al. 2007), the difficulty level of a three-back condition has been chosen to guarantee the cognitive demanding characteristic of this task. A familiarization phase including a detailed description and twelve stimuli was carried out prior to the experimental protocol at the same day. The outcome parameters were defined as mean response accuracy and mean reaction time. Non-responses were excluded from analysis and outcomes were calculated as the mean of all three trials.

Throughout the whole experimental procedure, EEG was assessed. The beginning and end of the single conditions were marked by setting automated timestamps using EEG recording software (Brain Vision Recorder, Brain Vision, Germany). After each set, RPE was assessed using the CR-20 Borg scale (Borg and Borg 2010).

### EEG recordings and analysis

Cortical activity was continuously recorded during the experiment utilizing a mobile EEG system (LiveAmp, Brain Products, Germany) with 64 active electrodes (actiCap, Brain Products, Germany). The electrode placement followed the standard international 10–20 system (Jasper 1958), with FCz as the reference and AFz as the ground electrode. The impedance level was kept below 25 kΩ. A wireless EEG amplifier (LiveAmp 64, Brain Products, Germany) was placed in a lightweight backpack carried by the participant to enable unrestricted motion. The sampling rate was set to 500 Hz.

For the data processing, EEG recordings were analyzed using the EEGLAB toolbox v2019 (Delorme and Makeig 2004) for MATLAB 2019b (Mathworks Inc., Natick, MA, USA). Relevant data was run through a preprocessing pipeline, starting with the Cleanline plugin (Mullen 2012) to remove line noise. Afterwards an finite impulse response band-pass filter from 3 to 30 Hz was applied and data were re-referenced to the common average as well as down-sampled to 256 Hz. Further, electrically bridged channels were removed using the eBridge tool (Alschuler et al. 2014) and an automatic rejection of artifact channels was applied based on deviations (> 5 SD) of the normalized power over the frequency range. After the pre-processing, time sections contaminated by non-stereotype artifacts were manually rejected across all channels. After artifact removal, an adaptive mixture independent component analysis (AMICA) algorithm (Palmer et al. 2011) was then applied to decompose EEG data into maximally independent components (ICs), allowing to separate sources of brain and non-brain electrical activity (Onton and Makeig 2006). By applying the DIPFIT plugin (Oostenveld and Oostendorp 2002), the approximate spatial source for all IC’s were calculated in a standardized realistic MRI model using the four-shell spherical head model (BESA, Germany). By means of the ICLabel plugin (Pion-Tonachini et al. 2019), functional brain components were labeled from the pool of ICs, and only those identified as ≥ 90% brain probability were included in the further analysis. From this sample, ICs with residual variance of ≥ 15.00% were excluded from the analysis (Onton and Makeig 2006). ICLabel and the residual variance of dipolar sources were used to support the objective identification brain components.

Furthermore, k-means clustering was performed based on the component equivalent dipole locations, scalp topographies, and power spectra within 2 standard deviations of the respective cluster. In line with our hypothesis, only the two clusters assigned to the frontal and frontocentral brain areas were considered for further analysis. The continuous data of each IC were then segmented into the three conditions (TT, EC, NB) and the mean power spectral density (PSD) in the theta frequency band (4–7.5 Hz) for each condition and each cluster was computed. Even if further frequency bands like the alpha or beta band are associated with sensorimotor control, we focus on attentional demands and the associated frontal theta frequency and excluded these frequency bands from analysis, as the demands in sensorimotor demands seem not comparable between open-skill and cardiovascular exercise as well as cognitive tasks.

### Statistics

All statistical analysis were run in SPSS Statistics (version 24.0, IBM Corporation, USA). Normal distribution was confirmed by applying the Shapiro–Wilk test and sphericity was confirmed by applying Mauchly's test. In case of violation of the sphericity assumption, Greenhouse–Geisser correction was applied. A one-way repeated measures ANOVA design with the factor condition (TT, EC, NB) was applied on EEG outcomes as well as RPE and HR values. The level of significance was set at *p* < 0.05. To detect differences between conditions, post-hoc paired *t* tests corrected for multiple comparisons (Bonferroni) were performed. For interpretation of main effects, partial eta square (*η*_p_^2^) was calculated, and considered as small (0.01), medium (0.06), or and large (0.14) effect sizes. In post-hoc comparisons, Cohen’s *d* was calculated, and interpreted according to benchmarks for small (*d* = 0.2), medium (*d* = 0.5) and large (*d* = 0.8) effects (Lakens 2013).

## Results

### Performance, heart rate and RPE

The mean response accuracy during NB was 84.33 ± 12.67% and the mean reaction time was 0.95 s (± 0.15). For TT, the analysis of IMUs revealed an approximated average number of 105.0 ± 6.0 hits per block (3 min).

The results of the one-way repeated-measures ANOVA revealed significant differences of HR (*F*_(2, 34)_ = 38.71, *p* < 0.01, *η*_p_^2^ = 0.70) and RPE (*F*_(1.51, 25.70)_ = 32.63, *p* < 0.01,* η*_p_^2^ = 0.66) in the three different conditions. Post-hoc analyses indicate a significant increase of HR from NB (77.1 ± 10.5) to TT (91.3 ± 14.0, *p* < 0.01, *d* = 1.52) and TT to EC (103.2 ± 15.2, *p* < 0.01, *d* = 0.98) and RPE from NB (7.6 ± 1.6) to TT (9.1 ± 2.0, *p* < 0.01, *d* = 1.38) and TT to EC (10.7 ± 1.1, *p* = 0.01, *d* = 0.84).

### Cortical activity

**Fig. 2:**
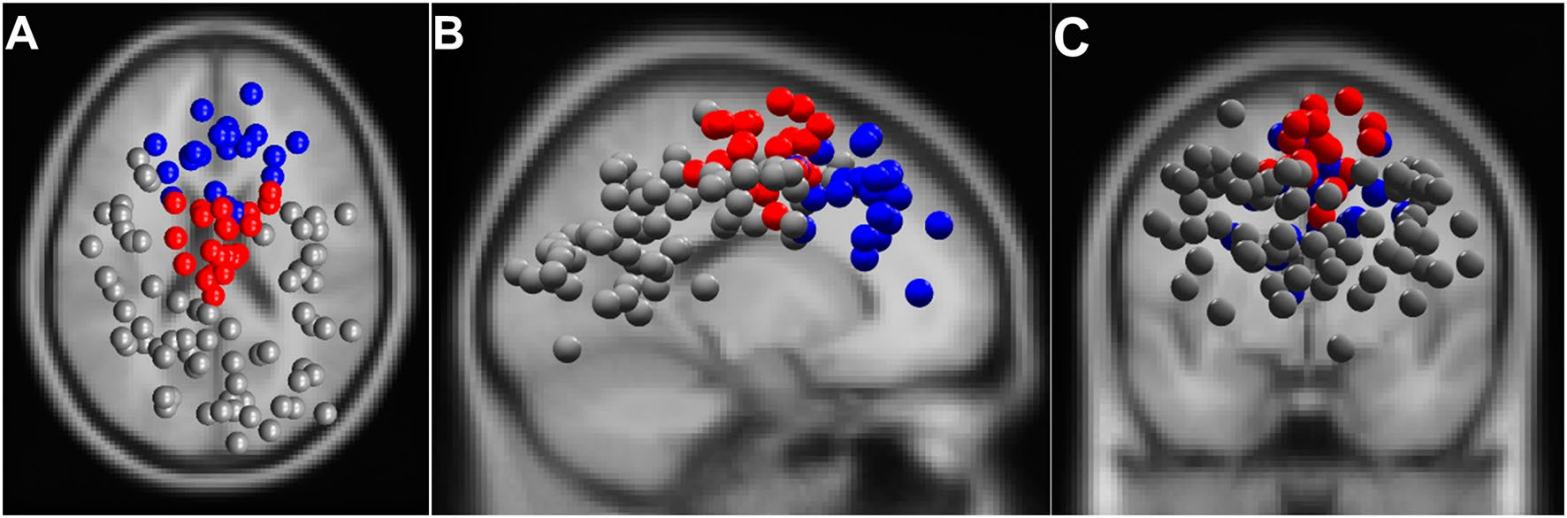
Approximated dipole localization of independent components (ICs) revealed from Adaptive mixture independent component analysis (AMICA) projected into a standard four-shell spherical head model (BESA, Germany). Dipoles are presented from **A** top, **B** sagittal and **C** coronal view. Dipoles marked blue are assigned to the frontal cluster (nIC = 21, subjects = 15, mean residual variance = 3.9 ± 2.7) and red dipoles are assigned to the frontocentral cluster (nIC = 21, subjects = 16, mean residual variance = 4.1 ± 2.2). Grey dipoles are assigned to clusters of functional ICs not further analyzed due to their topographical location in the brain

**Fig. 3:**
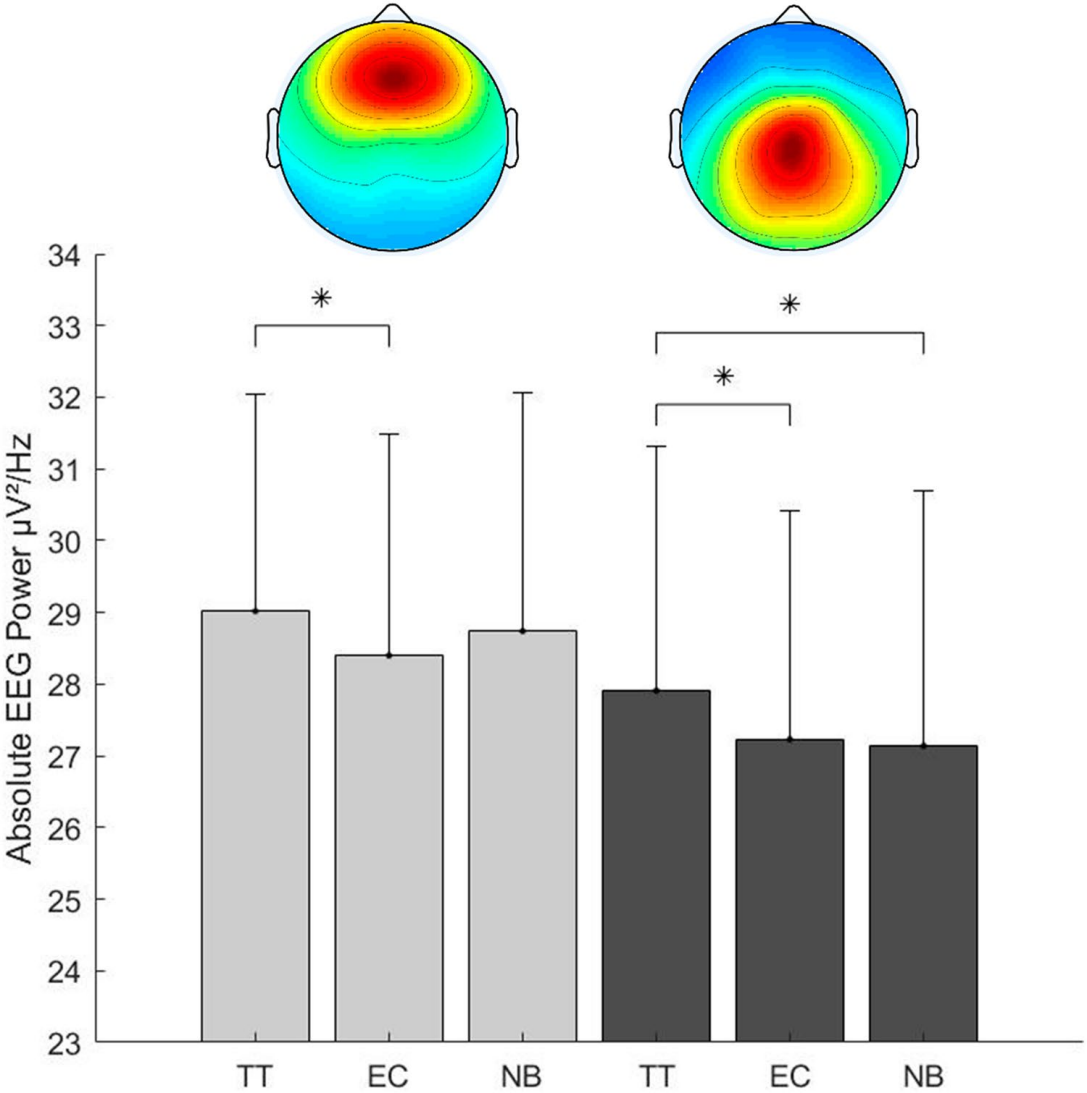
Absolute theta frequency power in µV^2^/Hz and scalp maps in the frontal (light grey bars) and central (dark grey bars) cluster during table tennis (TT), ergometer cycling (EC) and n-back task (NB). Significant differences resulting from pairwise comparisons corrected for multiple comparisons according to Bonferroni are indicated by a connecting line with respective asterisks; *p < 0.05

The k-means algorithm identified six cortical clusters of independent components assigned to the frontal, frontocentral, right motor, left motor, right parieto occipital and left parieto occipital cortex. The overall distribution of IC dipoles is displayed in Fig. 2. According to the hypothesis of the present research, the statistical analysis focused on the two clusters assigned to the frontal and frontocentral brain areas. Assignment to brain areas and description of mean RV, ICs included, subjects are presented in Fig. 2. The repeated-measures ANOVA revealed significant differences in theta power between the conditions in the frontal cluster (*F*_(2,40)_ = 10.65,* p* < 0.01, *η*_p_^2^ = 0.35) and the frontocentral cluster (*F*_(2,40)_ = 12.55,* p* < 0.01, *η*_p_^2^ = 0.39). Post hoc comparison using Bonferroni correction identified significant higher frontal theta power in TT (29.02 ± 3.04) compared to EC (28.4 ± 3.09, *p* < 0.01, *d* = 1.42). Theta power during NB (28.74 ± 3.33) in frontal brain areas did neither significantly differ from TT (29.02 ± 3.04, *p* = 0.23, *d* = 0.41) nor from EC (28.4 ± 3.09, *p* = 0.10, *d* = 0.50). Similar to the frontal cluster, the frontocentral cluster showed significant differences between the conditions. Post-hoc comparison revealed significant differences between the three conditions, excepting the comparison of EC (27.23 ± 3.19) to NB (27.12 ± 3.56, *p* = 1, *d* = 0.11). Theta power was significantly higher during TT (27.91 ± 3.41) compared to EC (27.23 ± 3.19, *p* < 0.01, *d* = 1.03) and NB (27.12 ± 3.56, *p* = 0.01, *d* = 1.06). The results in cortical activity are illustrated in Fig. 3.

## Discussion

The present study aimed to investigate activation of the frontal cortex in association with a TT play, an open skill sport characterized by an ever-changing and unpredictable environment. Therefore, theta activity in frontal brain areas was compared during continuous TT rallies with those observed during physical activity on the bicycle ergometer, a solely cardiovascular exercise and a cognitive working memory task devoid of physical activity. In accordance with our hypothesis, findings reveal that TT particularly induces increased activity in the prefrontal and frontocentral brain areas compared to cycling and a cognitive task.

In the present study, we measured EEG in participants playing continuous TT rallies. Therefore, this study is the first one demonstrating feasibility of assessing frontal cortex activity during continuous open-skill exercise. While previous EEG studies in open-skill sports restricted tasks to stationary repetitive movements (Skrzeba and Vogt 2018; Christie et al. 2019; Hülsdünker et al. 2020; Chuang et al. 2013), the present study did not analyze cortical activity associated with single movements, but rather focused on the continuous changes in cortical activity related attentional demands during unpredictable open-skill exercise using TT as a model. Thus, it seems to be feasible to get new insights into the cortical activity of various OS exercises and extent the understanding of open-skill sports as intervention (Gu et al. 2019) by measuring acute effects on the brain level using a mobile EEG device.

Cortical activity in the frontal cortex revealed significant differences in theta power between the three conditions. While TT and NB did not significantly differ in the frontal brain area, TT induced significant higher theta power than EC. The large effect size supports the assumption of differences in neural processing between EC and TT. Originally, frontal theta power in the area of the anterior cingulate cortex (ACC) has been associated with attentional demands (Sauseng et al. 2007). The state of focused attention and maintenance of task relevant processing has been related to an increase in theta power (Gevins et al. 1997; Gevins and Smith 2000; Smith et al. 1999; Hanslmayr et al. 2005; Sauseng et al. 2010). Therefore, it is suggested that the increase in frontal theta during TT compared to EC may be associated with higher demands in focused attention and task relevant processing. Since the NB also requires continuous cognitive processing (Scharinger et al. 2017) the lack of significant differences may indicate a comparable degree of attention between TT and NB.

In the context of cognitive control during exercise, an increase in frontal theta has also been related to error monitoring for maintenance of postural equilibrium as revealed by challenging postural control tasks (Hülsdünker et al. 2015). Further more dynamic movement studies supported the idea that theta power is involved in error monitoring and movement adaptation during sensorimotor tasks (Shirazi and Huang 2021; Sipp et al. 2013). The distinction between TT and EC regarding frontal brain activation might, therefore, be ascribed to the monitoring of relevant sensorimotor information to fulfill the performed task. Anders et al. (2018) demonstrated an increase in frontal theta power when increasing the complexity of information monitoring in an exergaming task requiring decision-making. Hereof, the unpredictability of the TT task potentially requires continuous decision making in a dynamic environment (Akpinar et al. 2012; Hülsdünker et al. 2020) and therefore increases task complexity compared to the well-predictable cardiovascular task. Accordingly, TT as an open-skill task may combine error monitoring and decision-making demands which may entail in increased theta power. Therefore, we assume that theta power in frontal brain areas might be an indicator of increased attentional demands in dynamic coordination exercise.

Next to the changes in the prefrontal cortex, we further identified a frontocentral cluster revealing increased theta power during TT compared to EC and the NB. Several EEG studies observed the presence of multiple frontal theta clusters during cognitive tasks (Töllner et al. 2017; Botvinick et al. 2004; Kerns 2006). Hereof, Töllner et al. (2017) suggested an increased representation of conflict-processing in frontocentral brain areas, since theta activity particularly increased when monitoring incongruent visual information. Further studies associated frontocentral brain activity with the reactive adjustment of neural processing to increase alertness in upcoming trials (Botvinick et al. 2004; Kerns 2006). TT as an open-skill sport is characterized by a substantial variability within consecutive trials (Akpinar et al. 2012; Guo et al. 2017) This variability goes along with demands in conflict processing and the ongoing reactive adjustments of movement to maintain a given rally. It might be suggested that the continuous reconciliation of multisensory information requires continuous conflict-related monitoring. Therefore, the involvement of frontocentral brain areas in open-skill sports such as TT possibly subserve a mechanism which allows the individual to maintain attention on the one hand and adequately adapt motor behavior throughout the exercise on the other hand.

For purposes of internal validation of increased attentional processing, frontal theta power was also investigated during a cognitive task. Even so. (Scharinger et al. 2017) suggested an increase in frontal theta power during NB, cardiovascular exercise and cognitive task did not differ significantly in this study. In the frontal cluster theta power demonstrated a trend towards significant increase compared to the EC condition. Considering the high precision rate during the NB task, (~ 84 ± 13%), but taking into account inter-individual differences in performance among participants, it might be possible that in some subjects the task was not challenging enough to induce significantly increased activation in frontal brain areas. In contrast, no significant difference was observed comparing frontocentral theta power between NB and EC. This might indicate a lack of conflict processing in both tasks due to predictability of task conduction.

The present study revealed increased attentional demands during open-skill exercise compared to cardiovascular exercise represented by an increase in frontal and frontocentral brain activity. Previous observational and interventional studies associated the beneficial effect of open-skill exercise on cognitive functions with increased demands in perception and processing of environmental stimuli (Gu et al. 2019; Ingold et al. 2020; Formenti et al. 2021). By demonstrating increased activity in frontal brain areas during acute open-skill performance, the present study may provide supporting evidence for increased information processing during open-skill exercise. Over a longer-period of time, the repetitive activation of frontal brain areas during open-skill exercise might be a potential driver of long-term neuroplastic changes in these brain regions (Guo et al. 2017). Therefore, open-skill sports are potentially effective interventions for maintaining and enhancing cognitive performances associated with attention and multisensory information processing. Particularly, population with deficits in these domains, such as elderly people, might be able to benefit from open-skill exercise interventions.

## Limitations

Some limitations of the present study need to be mentioned. As the EEG source space analysis only provides an approximation of the real cortical source, assignments of IC clusters to explicit brain structures should be avoided (Gramann et al. 2010). In line with this limitation, it needs to be mentioned that not all subjects contributed functional components to all clusters due to the blind source separation approach. This is a common problem for ICA-based EEG analysis resulting from blind-source separation and needs to be considered when analyzing the results (Huster et al. 2015). Therefore, the present investigation focused on broad frontal brain area rather than particular brain structures, so that the reconstruction of the origin of electrical activity provides a sufficient spatial resolution (Acar and Makeig 2013).

With regard to the functional interpretation of the observed theta increase, the complexity of TT play needs to be taken into account. Even if we suggest increased attentional demands due to decision making and movement adaptation, it remains open which specific mechanisms underlie the increase of frontal brain activation. For instance, visual perception and associated eye movements are mandatory during sensorimotor control and more evident during TT compared to the other conditions. Therefore, next to control of muscle contraction and decision making, gaze control, saccadic eye movements, and eye blinks may also induce activation of neural ensembles relevant for processing an internal sensory-motor representation and orientating in space (Cebolla and Cheron 2019; Nakano et al. 2013; Babapoor-Farrokhran et al. 2017; Wunderlich and Gramann 2021). Therefore, increased theta power in frontal brain areas might not only be a function of attentional demands related to the motor task, but also of conscious visual processing. Next to conscious eye-movement, postural control is another behavioral component that becomes relevant during adaptive, open-skill exercise, since individuals need to dynamically control their center of mass in space. Several studies indicated increased frontal theta activity during challenging postural demands (Gebel et al. 2020; Büchel et al. 2021; Hülsdünker et al. 2015). Thus, the complexity and multi-modality of open-skill exercise should always be considered when interpreting changes in functional brain outcomes. Especially for sports research, the investigation of gaze- and posture-induced cortical oscillations seems highly relevant to understand adaptive behavior in the real world. Future studies should unravel the contribution of the single components to frontal theta power by modulating existing open-skill exercises carefully.

Moreover, we focused on the frontal theta as this is associated with higher cognitive processes (Cavanagh and Frank 2014; Sauseng et al. 2010). However, it is important to note that a variety of brain areas and frequency bands can offer insights into the cortical processing in open-skill exercise. Parietal-occipital areas and hemispheric differences has been focused in visual processing of OS exercise (Hülsdünker et al. 2020) and alpha oscillations has also been related to in cognitive (Klimesch et al. 2007) and sensorimotor (Baumeister et al. 2008) aspects of human behavior. However, frontal theta is suggested to reflect the best holistic, integrative brain model of cognitive processing in open-skill exercise.

Even if this is the first study investigating brain activity during OS exercise, the ecological validity of the present study is still limited by instructing the participants to play in a cooperative manner. Since previous studies demonstrated that competition-induced emotions can affect EEG outcomes (Kim et al. 2017), the present findings need to be generalized with caution. Further, it needs to be mentioned that complex cognitive demands between NB, EC and TT cannot be directly compared with each other. However, all tasks were stated to affect the frontal cortex, so that we investigated this specific region for on-task differences in the activation of frontal cortex resources.

## Conclusions

The present study revealed exercise-specific patterns of frontal cortex activity comparing open-skill exercise with cardiovascular and cognitive exercise in healthy young adults. In particular frontal and frontocentral theta power increased during open-skill exercise due to continuous demands in perception and processing of environmental stimuli.

These findings may help to explore the beneficial effect of OS exercise on brain function and support the application of OS exercise for prevention and rehabilitation of cognitive function. Future studies need to investigate the acute and long-term neurophysiological responses to open-skill exercise in population with restrictions in cognitive control, attentional capacity or sensorimotor control. According to the present findings, TT may serve as a feasible intervention to support open-skill induced cortical activation in different populations, like elderly or children.

## Data Availability

The datasets generated during and/or analysed during the current study are available from the corresponding author on reasonable request.
